# Aqueous-Phase Reforming of Biogas Slurry over MOF-Derived α-MoO_3_ Catalyst for Producing Renewable Hydrogen: Effect of Fermenting Time

**DOI:** 10.3390/molecules29235565

**Published:** 2024-11-25

**Authors:** Qingguo Bu, Jian Wang, Yuxuan Chen, Junyu Tao, Akash Kumar, Beibei Yan, Guanyi Chen

**Affiliations:** 1 School of Environmental Science and Engineering, Tianjin University, Tianjin 300350, China; zjn8966@163.com (Q.B.); chen_yuxuan2018@126.com (Y.C.); akash.kumar@tju.edu.cn (A.K.); yanbeibei@tju.edu.cn (B.Y.); 2 China Energy Conservation and Environment Protection Engineering Co., Ltd., Beijing 100082, China; 3 School of Energy and Environment, Shenyang Aerospace University, Shenyang 110036, China; 4 School of Mechanical Engineering, Tianjin University of Commerce, Tianjin 300134, China; taojunyu@tjcu.edu.cn (J.T.); chengy@tjcu.edu.cn (G.C.); 5 School of Science, Tibet University, Lhasa 850012, China

**Keywords:** aqueous-phase reforming, MOF derivatives, biogas slurry, hydrogen, wastewater treatment

## Abstract

Aqueous-phase reforming (APR) is an alternative method for treating and utilizing biogas slurry (BS) to produce renewable hydrogen from organic oxygen-containing wastewater. Considering the fluctuating characteristics of BS with changes in the degree of fermentation, developing an efficient catalyst is a major concern for the APR of BS. The novel catalyst based on molybdenum-based metal–organic-framework-derived oxides (Mo-MOF-derived α-MoO_3_) was reported in this study. The results indicated that the variables (e.g., pH, organic load, and salinity) of BS corresponded to the fermentation times and exhibited decreasing trends after APR under the reaction conditions of 225 °C and 30 min. Decarboxylation was identified as the main side reaction in the APR of BS over the catalyst. An optimal yield of 2.17 mL_hydrogen_/mL_BS_ was achieved when BS was obtained from 6 days of fermentation. Finally, the Mo-MOF-derived α-MoO_3_ catalyst was obtained from the greater specific surface area of MOFs. The catalyst had a weaker acidity than the initial α-MoO_3_, making it more preferred for facilitating the APR of BS.

## 1. Introduction

Currently, large and medium-sized biogas projects are widely generalized and applied as carbon abatement technologies, especially in China. According to the Ministry of Agriculture and Rural Affairs, 1400 large-scale biogas projects were built between 2015 and 2017. These developments treat and utilize 6 billion tons of organic waste per year, such as straw, livestock manure, kitchen waste, and wastewater. However, the treatment and utilization of the resulting biogas slurry (BS) cannot be ignored [1]. On the one hand, the BS obtained from the solid–liquid separation of digestate is a large liquid fraction; on the other hand, although the fermentation of raw materials and treatment process significantly influence the characteristics of BS, BS is generally rich in large amounts of nutrients (e.g., nitrogen, phosphorus, and potassium), medium and micronutrients (e.g., Ca, Fe, Zn, and Cu), some microbial metabolites (e.g., amino acids, vitamins, active enzymes, hormones), and a large number of pathogens (e.g., Escherichia coli). This confirms that although BS can be sustainably used as a fertilizer or soil amendment, the potential for such sustainable BS consumption is small compared to expanding biogas projects. There are several restrictions on the carrying capacity of croplands and transportation costs [2]. If BS is returned directly to the field or used as a concentrated biogas fertilizer, it may result in environmental risks, such as water and land contamination [3,4,5]. Simultaneously, carbon-laden aqueous streams at the industrial level must be cleaned and priced to reduce the environmental and economic difficulties [6]. Therefore, it is essential to develop an alternative technology that can overcome the limitations of these mainstream methods to treat and utilize BS.

Aqueous-phase reformation (APR) is a catalytic process that can transform oxygenated compounds into H_2_-rich gases and chemicals under mild hydrothermal reaction conditions (200–250 °C, 1.5–5 MPa) [7,8]. Notably, most studies on APR have focused on individual oxygenated hydrocarbons, such as alcohols (methanol [9,10], ethanol [11], glycerol [12,13], etc.) and acetic acid [11,14]. Multi-component feedstocks or complex raw materials such as hydrogen carriers have a huge potential for large-scale hydrogen storage systems [15], especially green hydrogen [16]. Currently, APR is used to treat cheese whey wastewater [17,18,19], brewery wastewater [20,21], starch wastewater [22], and fruit juice [8], and it shows good performance in removing organic matter and generating H_2_. However, there is less research related to the above complex raw materials.

The selection and optimization of catalysts and reaction temperatures are crucial for the performance of APR. In terms of catalysts, the VIII family of transition metal-based catalysts is widely adopted because of the cleavage of the C-C bond. Pt-based catalysts with high activity are quasi-universal because of their high cost [6,23]. As for the temperature condition, the temperature is generally below 200–250 °C, which is relatively mild. The heating cost was further reduced by considering heat recovery. Consequently, if an appropriate non-noble metal catalyst is available, APR would be a more financially viable technique for treating BS.

In our previous reports [24], α-MoO_3_ nanosheets exhibited good performance in APR owing to their van der Waals (vdWs) heterostructures. However, the APR performance of α-MoO_3_ nanosheets is unsatisfactory because of their low surface area and non-porous structure. Various strategies have been employed to introduce mesopores into catalysts. Template methods using a surface-active agent are still in the development stage. Another method is alkali modification, which has been widely studied and exhibited good performance. However, these methods are expensive and require the addition of chemical reagents. Notably, metal-organic frameworks (MOFs) have unique properties, such as a rich pore structure and high surface area, and they have great potential as precursors to synthesize corresponding metal oxides through thermal treatment [25]. Therefore, Mo-MOF-derived α-MoO_3_ may be an effective catalyst for the APR of BS.

Most importantly, the APR of BS was determined by the degree of fermentation as it has a large influence on the physicochemical properties of BS, such as the pH, organic load, and salinity. Therefore, this study aimed to introduce a novel method that can simultaneously treat and utilize BS to assess the effect of the degree of fermentation and thus provide a reference. Specifically, this study developed a Mo-MOF-derived α-MoO_3_ catalyst and studied the catalytic performance of BS with the gradient of fermentation time.

## 2. Results and Discussion

### 2.1. Characterization of Catalysts

#### 2.1.1. Phase and vdW Heterostructures Analysis

**Figure 1:**
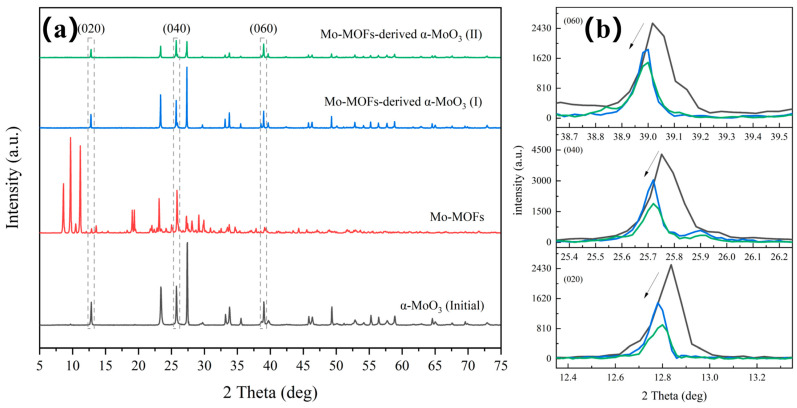
Phase and vdW heterostructures analysis of various catalysts: (**a**) XRD patterns and (**b**) specific XRD patterns around the diffraction peaks of (020), (040), and (060).

Figure 1a showed the XRD patterns of the Mo-MOF-derived α-MoO_3_ catalyst, which was the same as the simulated pattern [26]. This confirmed that the Mo-MOFs were successfully prepared in this study. Additionally, the diffraction peaks of the (020), (040), and (060) peaks of α-MoO_3_ shifted toward lower diffraction angles after treatment (Figure 1b). This was attributed to the presence of defects that alter the binding environment [27]. This also indicated that the vdW heterostructures of α-MoO_3_ obtained larger interspacing after the treatment [28,29], which can promote ion transport [30].

**Figure 2:**
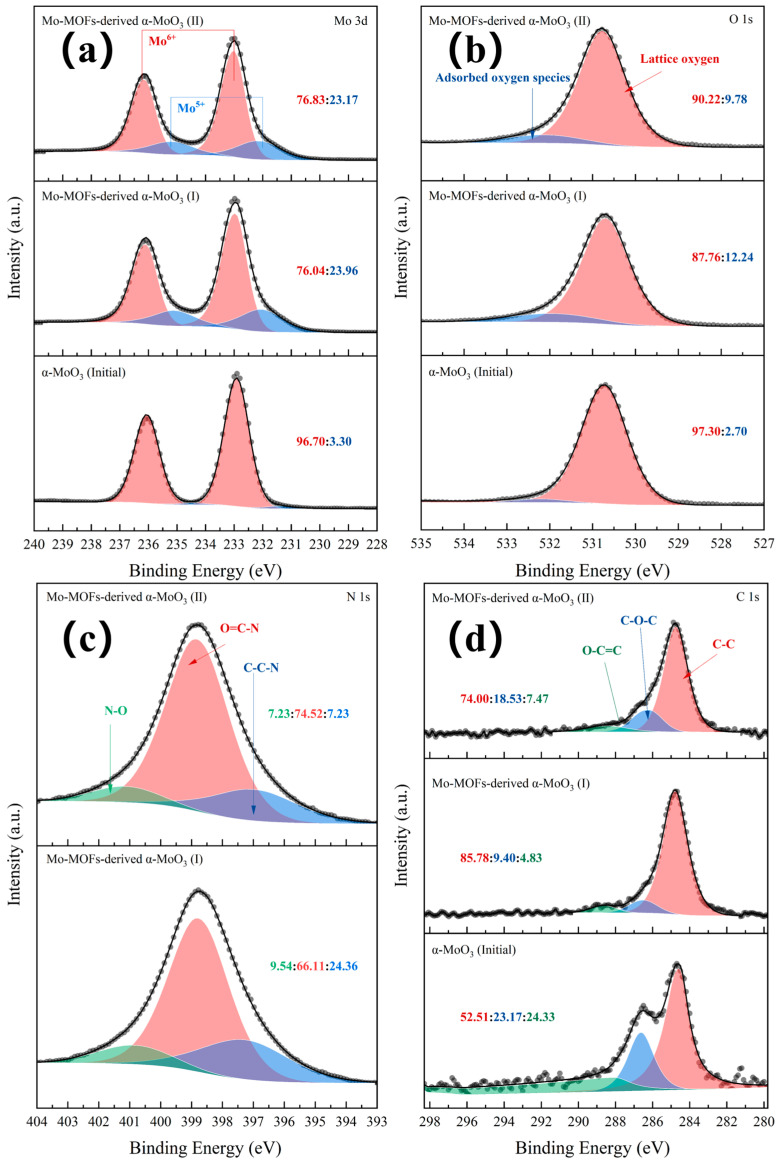
XPS patterns of various catalysts: (**a**) Mo 3d peak, (**b**) O 1s peak, (**c**) N 1s peak, and (**d**) C 1s peak.

The valence states of Mo, O, N, and C at different stages of the Mo-MOF-derived α-MoO_3_ catalyst were characterized by XPS (Figure 2). The initial catalysts contained a full fraction of Mo^6+^ species as α-MoO_3_. After the treatment, Mo^5+^ species appeared in the α-MoO_3_ nanosheets (Figure 2a). The peaks at 530.3 eV and 532.90 eV were attributed to the lattice and chemisorbed oxygen (Figure 2b). The N species mainly involved the N, O=C-N, and N-O groups [31]. This indicated that imidazole-derived species existed in the catalysts (Figure 2c). The peak at 284.55 eV was attributed to the α carbon atom (e.g., C-C, C-H, or C-C), whose content was the greatest (Figure 2d). The presence of N and C species can be attributed to the loss of organic ligands and structure collapses after the high-temperature calcination of MOFs. Figure S1 displays the oxygen vacancies that were present in α-MoO_3_ before and after the treatment. Moreover, the content of oxygen vacancies of α-MoO_3_ (initial) was higher than that of Mo-MOF-derived α-MoO_3_ (I) and (II) catalysts. Among them, the content of oxygen vacancies of Mo-MOF-derived α-MoO_3_ (I) was slightly higher than that of Mo-MOF-derived α-MoO_3_ (II). This suggested that Mo-MOF-derived α-MoO_3_ (II) had the strongest oxidation. Small amounts of oxidants in a hydrothermal system are beneficial for the decomposition of organic matter to produce H_2_ [32]. Therefore, Mo-MOF-derived α-MoO_3_ (II) had more potential to be used as a catalyst for the APR of BS.

#### 2.1.2. Surface Property Analysis

**Figure 3:**
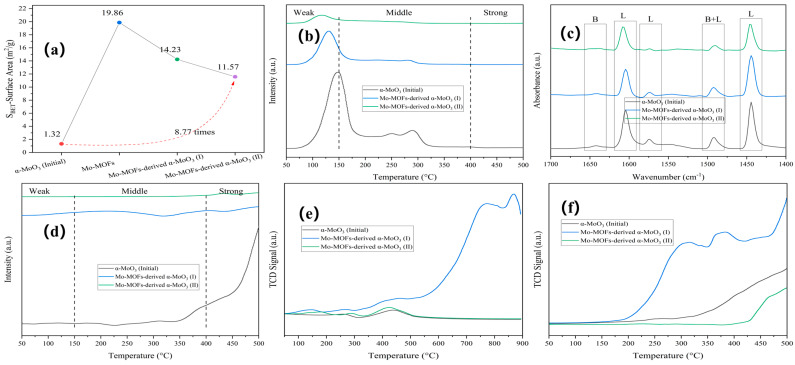
Characteristics of various catalysts: (**a**) BET, (**b**) NH_3_-TPD, (**c**) Py-IR, (**d**) CO_2_-TPD, (**e**) O_2_-TPO, and (**f**) H_2_-TPR.

Rod-shaped Mo-MOFs with smooth surfaces were synthesized by a hydrothermal method using a Mo precursor (Figure S2). The rod-shaped Mo-MOF-derived α-MoO_3_ was fractured and agglomerated following calcination and thermal hydrogenation. Mo-MOF-derived α-MoO_3_ (II) showed a 8.77-fold increase in specific surface area (Figure 3a).

The acid–base property of the catalyst surface is critical for understanding the catalytic reaction behavior [33], especially in APR. For the weak, medium, and strong sites, the temperature ranges of the desorption peaks are 30–150 °C, 150–400 °C, and >400 °C, respectively [34]. As shown in Figure 3b, weak and medium acid sites were observed in α-MoO_3_ (initial). After calcination and thermal hydrogenation of the Mo-MOFs, the intensity of the weak sites was greatly diminished, and the desorption peak temperature exhibited a lower shift. As shown in Figure 3c, the type and number of acid sites were accurately characterized by Py-IR. Considering the two-step procedure of the catalysts that was related to calcination and thermally hydrogenating, the order of total acidity for the catalyst was α-MoO_3_ (initial) > Mo-MOF-derived α-MoO_3_ (I) > Mo-MOF-derived α-MoO_3_ (II), as shown in Table S1. This confirmed that the surface acidity was reduced after calcination and thermal hydrogenation, which can inhibit carbon deposition and improve catalytic activity during APR [33]. The total basicity of the catalysts followed the same order (Figure 3d and Table S1).

The redox properties of the catalysts were investigated by using O_2_-TPO and H_2_-TPR. The results are shown in Figure 3e,f. α-MoO_3_ (initial) showed a peak for surface-adsorbed oxygen around 250 °C and a peak for lattice oxygen around 450 °C [35]. After the calcination of Mo-MOFs, there was a peak at around 150 °C, and the intensity of Mo-MOF-derived α-MoO_3_ (I) was higher than of α-MoO_3_ (initial). The results showed that the introduction of the imidazole-derived species enhanced the oxidability of the catalysts. Mo-MOF-derived α-MoO_3_ (II) had lower peak intensities than Mo-MOF-derived α-MoO_3_ (I), indicating that thermal hydrogenation can enhance the oxidize resistance. Furthermore, the O_2_-TPO results of Mo-MOF-derived α-MoO_3_ (II) indicated that oxidation began slowly at 175 °C. This indicated that the oxygen vacancies of Mo-MOF-derived α-MoO_3_ (II) were stable at a reaction temperature of 225 °C. These results are consistent with the XPS results (Figure 2). At the same time, H_2_-TPR proceeded in the following order: Mo-MOF-derived α-MoO_3_ (I) > Mo-MOF-derived α-MoO_3_ (II) > α-MoO_3_ (initial). This meant that Mo-MOF-derived α-MoO_3_ (II) had a greater reducing resistance, which was favorable in H_2_ evolution reactions. Considering that moderate redox reactions were conducive to enhancing the performance of APR for H_2_ generation, Mo-MOF-derived α-MoO_3_ (II) was used as a catalyst in the APR of BS in this study.

### 2.2. Pollutant Treatment Capability

Salinity and organic load are important water quality indicators. The TDS represents the total amount of dissolved organic and inorganic solids. The EC reflects the amount of inorganic salt. COD represents the amount of reducing substances, and NPOC reflects the overall amount of organic matter. Additionally, AN describes free NH_3_ and NH^+^_4_ in water. In the study, the above indicators were adopted to study the Mo-MOF-derived α-MoO_3_ (II) catalysts on the APR performance of BS.

**Figure 4:**
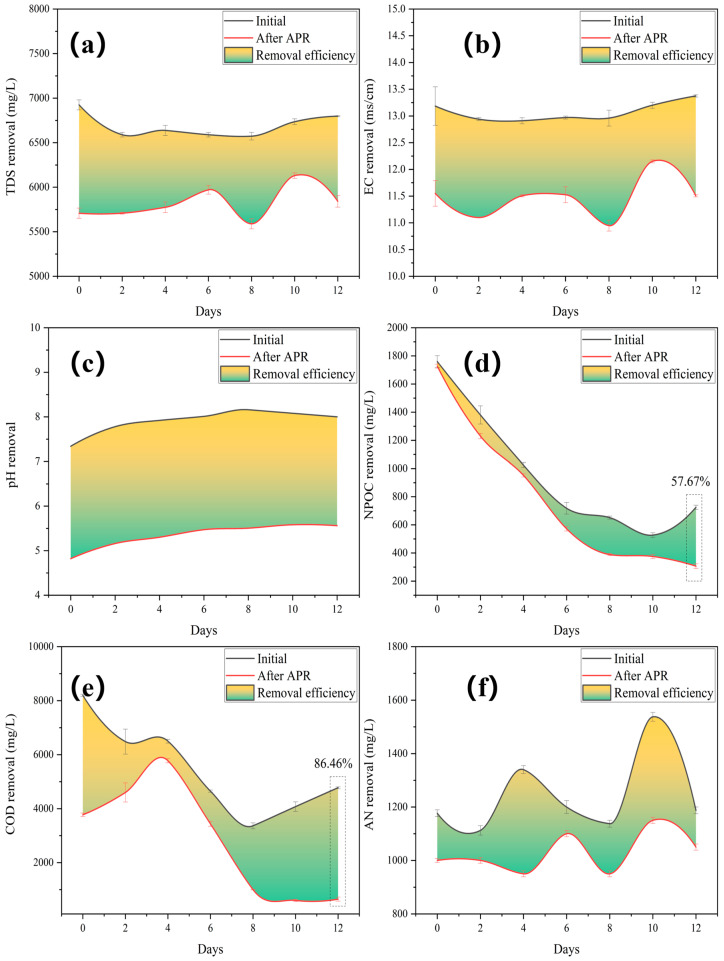
Removal performances after treating gradient BS with Mo-MOF-derived α-MoO_3_ (II) catalysts: (**a**) TDS, (**b**) EC, (**c**) pH, (**d**) NPOC, (**e**) COD, and (**f**) AN (reaction conditions: 225 °C, 30 min, 1 g catalyst, and 50 mL BS).

The TDS and electrical conductivity (EC) were significantly reduced by the addition of the catalyst (Figure 4a,b). This implied that the dissolved solids underwent a phase transition from liquid to solid. The pH increased gradually after the fall and peaked after 8 days of fermentation (Figure 4c), indicating that hydrolytic acidification played a dominant role in the accumulation of organic acids, leading to a decreased pH. This further demonstrated that the 8-day fermentation period was the crucial interval separating the fermentation stage. Insoluble organic matter macromolecules are broken down into small molecules of water-soluble low fatty acids during the first step of anaerobic hydrolysis. The second step involves the generation of anaerobic acid. At this stage, fermentation bacteria convert low fatty acids into H_2_, formic acid, etc., which makes BS acidic. Simultaneously, the application of the catalyst affected the pH, and BS changed from being slightly alkaline to being acidic.

The NPOC removal efficiency increased with a decrease in the initial NPOC (Figure 4d). The AN and COD removal efficiencies showed the same decreasing and then increasing trends (Figure 4e,f), which were affected by the fermentation stage. The generation of oxygen vacancies was caused by the release of lattice oxygen, which served as an oxidant for COD removal. Superoxide radicals are formed on oxygen vacancies, which can induce organic aerobic coupling between amines and their corresponding imines [36]. Additionally, despite the fluctuations in water quality, the highest values for NPOC (57.67%) and COD (86.46%) were obtained.

### 2.3. Hydrogen Production

**Figure 5:**
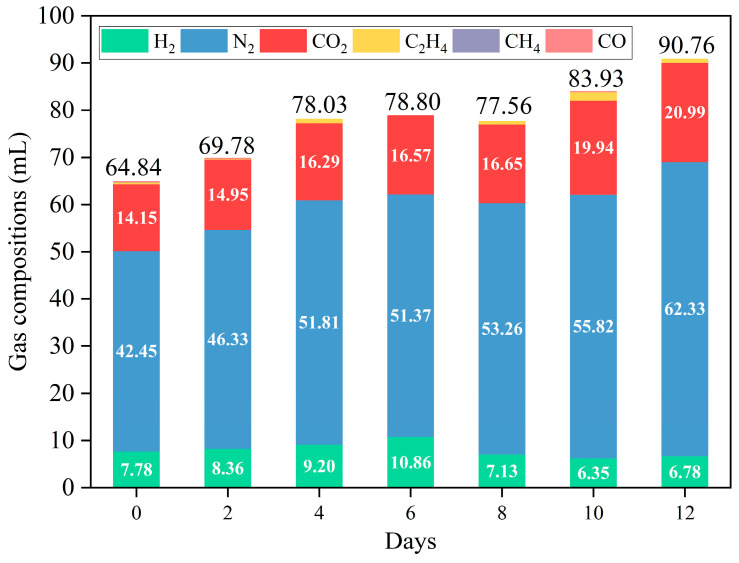
Effect of APR without/with catalysts on the volume and composition of the gas (reaction conditions: 225 °C, 30 min, 1 g catalyst, and 50 mL BS).

The volume and composition of the gases are shown in Figure 5 and Table S2, with H_2_, N_2_, and CO_2_ being the primary gases. The yield of H_2_ first decreased and then increased, reaching up to 10.86 mL when fermentation was over six days. Additionally, H_2_ content can reach 7.47 vol%–13.78 vol% (Table S2), similar to the results of other studies (Table S3). In addition to shielding, denitrification also produces N_2_. Overall, the yield of N_2_ and CO_2_ increased with prolonged fermentation. The yield of N_2_ decreased slightly after 6 days of fermentation compared to that after 4 days of fermentation. This may be due to thermal hydrolysis [37] and hydrothermal carbonization [8,38], which resulted in dehydrogenation in the presence of carbon (coke) [39]. This raised the notion that the variation in gas production was caused by dissolved salts in BS. On the one hand, to a certain extent, the evaporation rate of organic compounds is reduced owing to inorganic salts in aqueous solutions, which tended to form carbon (coke) [19]. On the other hand, SiO_2_ and other metallic oxides (e.g., Al_2_O_3_, CaO, Fe_2_O_3_, MgO, and FeO), which have dehydrogenating abilities, were formed [40]. Meanwhile, CO_2_ can react with alkaline media, reducing CO_2_ content. This corresponded to the results of the NPOC removal efficiency (Figure 4). Notably, the ratio of CO_2_ to H_2_ was lower than usual in APR. This may be attributed to the denitrification and H_2_ production of amides during APR [24].

C_2_H_4_ was commonly present in the gas generated from the APR of BS at different fermentation times, except at 6 days of fermentation (Figure 5 and Table S2). This suggests that Fischer–Tropsch synthesis, which consumes H_2_ to produce alkenes instead of alkanes, may also occur (Equation (1)). This can be attributed to the acidic sites in the catalyst [41,42]. The main reactions in Fischer–Tropsch synthesis (1) are as follows:

Produced alkanes: nCO + (2n+1)H_2_ → C_n_H_2n+2_ + 2H_2_O, ΔH > 0 Produced alkenes: nCO + 2nH_2_ → C_n_H_2n_ + nH_2_O, ΔH > 0 (1)

In summary, the APR of BS not only removes organic matter but also generates H_2_. Based on the result of pollutant treatment capability (Section 2.2) and H_2_ production (Section 2.3), Mo-MOF-derived α-MoO_3_ (II) can be a potential catalyst for the APR of BS compared to other studies, as shown in Table S3.

### 2.4. Compositions of Liquid Phase

#### 2.4.1. Organic Components

**Figure 6:**
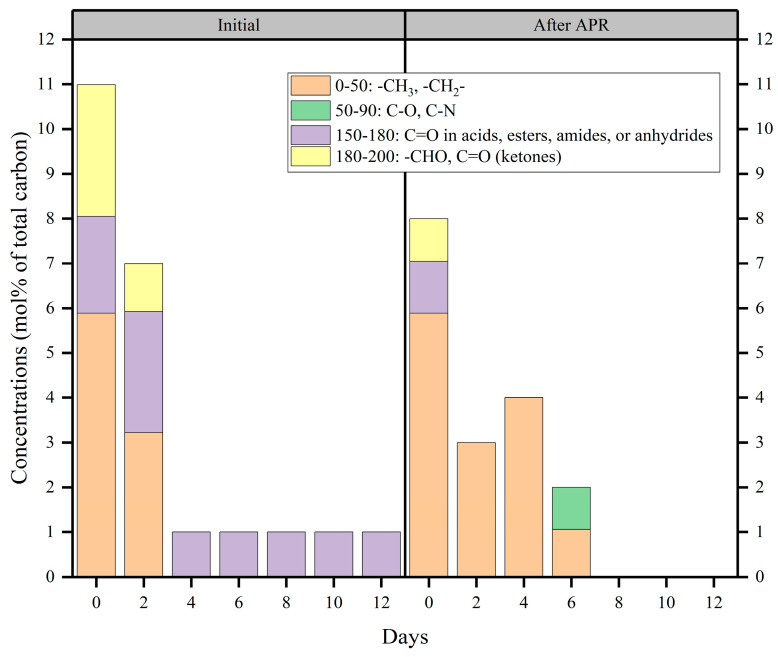
^13^C-NMR chemical shift assignment ranges and carbon contents of the liquid treatment obtained from treatment of gradient BS via Mo-MOF-derived α-MoO_3_ (II) catalysts (reaction conditions: 225 °C, 30 min, 1 g catalyst, and 50 mL BS).

To further understand the effect of fermentation days on the APR of BS, the organic components of BS before and after APR were investigated by ^13^C-NMR based on functional groups (Figure 6 and Figure S3). The chemical shifts at 0–50 ppm are related to the alkyl chains. The chemical shifts at 50–90 ppm indicate the replacement of aliphatic carbon by oxygen and nitrogen. When the chemical shifts were in the ^13^C-NMR spectral range of 150–180 ppm, they mainly represented the carboxylic carbon group. This indicated the presence of acids, esters, amides, or anhydrides. Additionally, the chemical shifts at 180–200 ppm reflect the carboxylic carbon in aldehydes and ketones [43,44].

As shown in Figure 6, initial BS from fermentation at 0 and 2 days had much richer carbon-containing functional groups, indicating that the distiller grains underwent anaerobic hydrolysis to produce more substances, such as amino acids, fatty acids, and saccharides. This consisted of the results presented in Section 2.2. In addition, the degree of anaerobic fermentation increased with the number of fermentation days. Initial BS contained more carbonyl compounds, such as esters and amides. The concentration of alkyl functional groups was unaffected by applying catalysts to the initial BS produced by fermentation on days 0 and 2. Moreover, the carbonyl functional groups were completely converted into alkyl, C-O, and C-N functional groups upon treatment with the original BS produced after 4 and 6 days of fermentation. Beyond 6 days of fermentation, the carbon-containing functional groups were completely converted to non-carbonaceous functional groups in the liquid phase. According to the results, it could be confirmed that Mo-MOF-derived α-MoO_3_ (II) catalysts mainly contributed to the carbonyl functional groups. Figure 6 shows that acids, esters, amides, and anhydrides prevailed during fermentation. Previous reports [24] have also indicated that amides are abundant in BS. These results prove that N_2_ and H_2_ are mainly derived from amides during the APR of BS. Additionally, the catalytic system was accompanied by a decarbonylation reaction. This was because carbonyl compounds, such as large amounts of carboxylic acids, accumulate during the anaerobic acidogenic stage. This also explained that NPOC removal efficiency increased with a decrease in the initial NPOC (Figure 4d). Hence, the carbonylation of carbonyl compounds was a decarboxylation reaction. Therefore, it immediately produced more CO_2_ than H_2_; this was why CO_2_ is produced in much larger quantities than H_2_. This confirmed the results presented in Section 2.3. The volume of CO_2_ generated was much higher than H_2_ due to the main causes that were outlined.

#### 2.4.2. Inorganic Components

**Figure 7:**
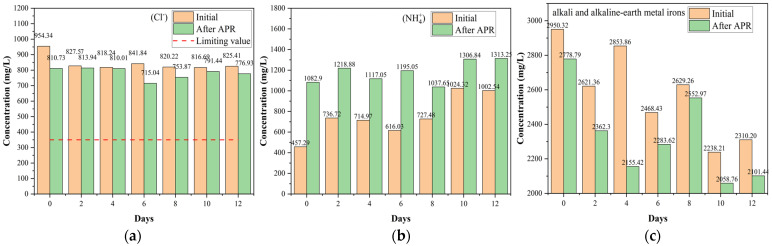
Effect of catalysts on the ion concentration (mg/L): (**a**) Cl^−^, (**b**) NH_4_^+^, and (**c**) alkali and alkaline earth metal irons (reaction conditions: 225 °C, 30 min, 1 g catalyst, and 50 mL BS).

The inorganic components of BS affect catalytic performance and increase the risk of returning farmland. Although a direct return to farmland is the most beneficial, it still faces many problems, such as potential water, food, and land risks. Following the irrigation water quality regulations (National Standards of China GB 5084-2021) in this study, hazardous ions and heavy metals were classified and analyzed [45]. F^−^ and Cl^−^ are hazardous ions, whereas the others are non-hazardous (Figure 7 and Figure S4). F^−^ ions were not detected, and the amount of Cl^−^ slightly decreased (Figure 7a). At the same time, the NH_4_^+^ content considerably increased owing to the acidic conditions (Figure 7b). In addition, NH_4_^+^ is representative of inorganic nitrogen. The total alkali and alkaline-earth metal orons contents decreased, as shown in Figure 7c. There was a slight competitiveness in alkali and alkaline earth metal removal, indicating that alkali and alkaline earth metal irons containing catalysts exhibited significant H_2_ production and heavy metal removal from the liquid phase during the hydrothermal reaction process [46]. This can be attributed to the heavy metals attached to the surface of the catalysts owing to oxidation and hydration, which affected the activation of the catalyst. Furthermore, Figure 7c confirmed that alkali and alkaline earth metal ions were considerably present in BS. Notably, alkali and alkaline earth metal ions, such as K [47], are self-catalysts that strengthen H_2_ production and organic substrate conversion, affecting the performance of the ex-catalyst during APR. Considering the results of the pollutant treatment capability (Section 2.2) and H_2_ production (Section 2.3), it can be inferred that inorganic salts have an important influence on the APR behavior of BS, especially alkali and alkaline earth metals. However, de Miera et al. [8] found that salinity had a detrimental impact on the yield of H_2_ at high salinities, most likely because of catalyst deactivation and the increased contribution of hydrothermal carbonization. They also found that salinity did not affect COD removal and total organic carbon within the tested range. In terms of organic matter removal, the results of this study were slightly different from those of Saenz de Miera et al. [8]. This can be attributed to the complexity and content of the inorganic salts.

Considering that BS became acidic and that the total content of alkali metals and alkaline earth metals decreased after APR, heavy metals were precipitated by oxidation and hydration, resulting in lower levels. Finally, as illustrated in Figure S5, the number of various types of heavy metals in BS decreased after implementing APR, reducing pollution risks.

## 3. Materials and Methods

### 3.1. Materials

Gradient BS was separated and obtained after distiller grains underwent anaerobic fermentation. Chemicals used in this study, including deionized water, molybdenum trioxide (MoO_3_, 99%, MERYER, Shanghai, China), and imidazole (99%, HEOWNS, Tianjin, China), were of analytical grade.

### 3.2. Preparation of the Catalysts

The following two-step procedure was used to prepare the Mo-MOF-derived α-MoO_3_. (1) In the synthesis of Mo-MOF-derived α-MoO_3_, the preparation process was as follows: 9.49 g Imidazole and 20 g α-MoO_3_ were dissolved in 100 mL of deionized water. The obtained slurry was transferred into a 150 mL sealed Teflon-lined autoclave, heated to 150 °C and held for 24 h to ensure complete crystallization. To synthetize Mo-based MOFs, the slurry was rinsed with 100 mL of deionized water and dried at 50 °C. To obtain Mo-MOF-derived α-MoO_3_ (I), the as-prepared product was calcined at 600 °C for 1.5 h with an airflow of 500 N mL/min and heating rate of 20 °C/min. (2) Thermal hydrogenation was carried out to obtain a hybrid material formed by α-MoO_3_ nanosheets. The synthesis details are provided in the Supplementary Materials, according to a previous study [24]. The sample was combined with 100 mL of a 30% H_2_O_2_ aqueous solution in a beaker and allowed to sit for 48 h. The solution was then mixed with 50 mL of aqueous saccharose solution (317.08 g/L) and 100 mL of distilled water. Saccharose consumes excess H_2_O_2_, with a molar ratio of C to Mo of 4. The suspension was dried at 80 °C and stirred until it was completely dry. All samples were dried for 24 h at 105 °C. To obtain Mo-MOF-derived -MoO_3_ (II), the samples were calcined at 600 °C for 1.5 h under a 500 N mL/min airflow and 20 °C/min heating rate.

### 3.3. Anaerobic Digestion Process

An anaerobic fermentation experiment was performed using the discard method. A digester containing 30 g of distiller grains and 800 mL of inoculum was placed in an incubator at 37 °C for anaerobic fermentation. Based on preliminary experiments on anaerobic fermentation (0–12 days), seven groups were devised to enable BS collection every 2 days [48].

### 3.4. Aqueous-Phase Reforming Experiments

The autoclave (100 mL) was made of stainless steel and was used to perform the APR of BS. BS was centrifuged for 5 min at 10,000 rpm before testing. Following centrifugation, BS was clarified to remove impurities that were not visible to the naked eye. Typically, as shown in Figure S6, the reactor was filled with 50 mL of BS and 1 g of a catalyst. Subsequently, the air was removed from the reactor using a high-purity argon purge. The reaction was conducted at 225 °C with autogenous pressure (2.5 MPa) and stirred at 200 rpm for 30 min. The ideal gas equation was used to determine the volume of gases under typical circumstances. Additionally, the initial pressure was 0 MPa when argon was incorporated as a purge gas; thus, the gas pressure after the reaction represents the pressure of all the generated gases. Finally, vacuum filtering was utilized to obtain the liquid and catalysts that were used.

### 3.5. Data Analysis

The catalytic performances were calculated as follows:

Gases’ yield = gases’ volume × gases’ content × 100%(2)

(3) $$\mathrm{TDS},\;\mathrm{EC},\;\mathrm{pH},\;\mathrm{NPOC},\;\mathrm{COD},\;\mathrm{and}\;\mathrm{AN}\;\mathrm{removal}\;\mathrm{efficiency}\;\;\left(\%\right)=\frac{X_{\mathrm{initial}\;\mathrm{solubility}\;(\mathrm{mg}/L)}-X_{\mathrm{finial}\;\mathrm{solubility}\;(\mathrm{mg}/L)}}{X_{\mathrm{initial}\;\mathrm{solubility}\;(\mathrm{mg}/L)}}\times 100\%$$

where TDS, EC, NPOC, COD, and AN represent the total dissolved solids, electrical conductivity, non-purgeable organic carbon, chemical oxygen demand, and ammonia nitrogen, respectively. X denotes the value of the six indices.

### 3.6. Analytical Methods

The surface elements, oxygen vacancies, microstructures, and surface areas of the catalysts were determined using X-ray powder diffraction (PANalytical, Almelo, The Netherlands), X-ray photoelectron spectroscopy (XPS, Thermo Scientific Escalab 250Xi, Waltham, MA, USA), electron paramagnetic resonance (EMXPlus-10/12, Bruker), scanning electron microscopy (SEM, JEOL, Tokyo, Japan), and the Brunauer–Emmett–Teller method (Micrometric Acusorb 2100E apparatus, Ottawa, ON, Canada).

Carbon deposition, reducibility, and acidic and basic characteristics of the catalysts were evaluated using O_2_-TPO, H_2_-TPO, NH_3_-TPD, and CO_2_-TPD (CHEMBET-3000 Chemisorption Instrument, Boynton Beach, FL, USA), respectively. The acid characteristics were evaluated using pyridine adsorption Fourier transform infrared spectroscopy (Thermo Fisher Nicolet 6700, Waltham, MA, USA).

Salinity (TDS and EC, METTLER TOLEDO, FiveEasy Plus, Hong Kong, China), pH (PHS-3C, INESA Scientific Instrument Co., Ltd., Shanghai, China), COD (DRB200/DR900, HACH, USA), AN (DRB200/DR900, HACH, Loveland, CO, USA), TN (DRB200/DR900, HACH, Merlin, OR, USA), and NPOC (TOC-L CPH/CPN, SHIMADZU, Kyoto, Japan) were also used in this study.

The species and concentrations of the gases were determined using gas chromatography (Agilent 7890A, Agilent, Santa Clara, CA, USA). A liquid nuclear magnetic resonance instrument was used with ^13^C spectra (AV 400, Bruker Inc., Fällanden, Switzerland), an ion chromatography instrument (Thermo Dionex, Sunnyvale, CA, USA), and inductively coupled plasma mass spectrometry (Thermo Scientific iCAP Q, Waltham, MA, USA) to determine the detailed functional groups, ions, and element content in the APR of BS, respectively.

The Supplementary Materials include information regarding the aforementioned analytical methods and a previous study [49].

## 4. Conclusions

BS was simultaneously treated and utilized to produce H_2_ through the APR over the Mo-MOF-derived α-MoO_3_ catalyst. This study revealed that fermentation time is a crucial parameter in the APR of BS owing to perturbations in salinity, pH, and organic load. Additionally, Mo-MOF-derived α-MoO_3_ (II) as the catalyst can promote APR compared with commercial α-MoO_3_ due to its decreased acidity and increased specific surface area. Meanwhile, decarboxylation, as a side reaction, is highly significant and contributes to a decrease in H_2_ purity. This study provides valuable technical references for wastewater treatment and renewable H_2_ production. Additionally, while MOF derivatives have good surface area and structure, MOFs’ commercial production is in their infancy.

## Data Availability

The raw data supporting the conclusions of this article will be made available by the authors upon request.

## Supplementary Materials

The following supporting information can be downloaded at: https://www.mdpi.com/article/10.3390/molecules29235565/s1, Figure S1: EPR patterns of various catalysts; Figure S2: SEM of various catalysts; Figure S3: 13C-NMR chemical shift assignment ranges and carbon contents of the liquid obtained from gradient BS before and after treatment with Mo-MOFs-derived α-MoO3 (II) catalysts (Reaction conditions: 225 °C, 30 min, 1 g catalyst, and 50 mL BS); Figure S4: Effect of catalysts on the ion concentration (mg/L) (Reaction conditions: 225 °C, 30 min, 1 g catalyst, and 50 mL BS); Figure S5: Influence of the treatment without/with catalysts on the heavy metals (μg/L): (a)~(g) the hazardous heavy metals and (h)~(m) the non-hazardous heavy metals (Reaction conditions: 225 °C, 30 min, 1 g catalyst, and 50 mL BS); Figure S6: Experimental procedure for the APR of BS; Table S1: Distribution of calculated acidic-basic sites and B/L ratio based on Py-IR and CO_2_-TPD of catalysts (μmol/g); Table S2: Gases composition, volume and pressure obtained from APR of BS at different fermentation days (Reaction conditions: 225 °C, 30 min, 1 g catalyst, and 50 mL BS); Table S3: Performance comparison of main characterization and results of APR of biomass-based organic wastewater; Table S4: The volume of BS after APR with different fermentation days (Reaction conditions: 225 °C, 30 min, 1 g catalyst, and 50 mL BS). References [17,18,24,30,38,50,51] appear in the Supplementary Materials.
