# Supplementary material for: Aqueous-Phase Reforming of Biogas Slurry over MOF-Derived α-MoO3 Catalyst for Producing Renewable Hydrogen: Effect of Fermenting Time
Source: Molecules. 2024 Nov 25;29(23):5565. doi: 10.3390/molecules29235565 (PMC11643699; doi:10.3390/molecules29235565)
Supplement: Supplementary file 1 [file molecules-29-05565-s001.zip › molecules-3214198-supplementary.pdf]

Aqueous-phase reforming of biogas slurry over MOFs-derived α-MoO_3 catalyst for producing renewable hydrogen: Effect of fermenting time

Qingguo Bu [1,5], Jian Wang [2,*], Yuxuan Chen [1], Junyu Tao [3], Akash Kumar [1], Beibei Yan [1], Guanyi Chen[3,4]

1. School of Environmental Science and Engineering, Tianjin University, Tianjin 300350, China;

2. School of Energy and Environment, Shenyang Aerospace University, Shenyang Aerospace University, Key Laboratory of Clean Energy, Shenyang 110036, China;

3. School of Mechanical Engineering, Tianjin University of Commerce, Tianjin 300134, China;

4. School of Science, Tibet University, Lhasa 850012, China;

5. China Energy Conservation (Beijing) Energy Conservation and Environment Protection Engineering Co., Ltd.

***Corresponding Author:** Jian Wang. E-mail: wangjian2023@email.sau.edu.cn

Contents

***Corresponding Author:** Jian Wang. E-mail: wangjian2023@email.sau.edu.cn................1

Figure S1. EPR patterns of various catalysts..3

Figure S2. SEM of various catalysts..4

Table. S1. Distribution of calculated acidic-basic sites and B/L ratio based on Py-IR and CO_2-TPD of catalysts (μmol/g)...5

Table. S2. Gases composition, volume and pressure obtained from APR of BS at different fermentation days (Reaction conditions: 225 °C, 30 min, 1 g catalyst, and 50 mL BS)......6

Table. S3. Performance comparison of main characterization and results of APR of biomass-based organic wastewater 7

Table. S4. The volume of BS after APR with different fermentation days (Reaction conditions: 225 °C, 30 min, 1 g catalyst, and 50 mL BS)..12

Figure S3. ^{13}C-NMR chemical shift assignment ranges and carbon contents of the liquid obtained from gradient BS before and after treatment with Mo-MOFs-derived α-MoO_3 (Ⅱ) catalysts (Reaction conditions: 225 °C, 30 min, 1 g catalyst, and 50 mL BS)..................14

Figure S4. Effect of catalysts on the ion concentration (mg/L) (Reaction conditions: 225 °C, 30 min, 1 g catalyst, and 50 mL BS)...15

Figure S5. Influence of the treatment without/with catalysts on the heavy metals (μg/L): (a)~(g) the hazardous heavy metals and (h)~(m) the non-hazardous heavy metals (Reaction conditions: 225 °C, 30 min, 1 g catalyst, and 50 mL BS)...16

2.2. Preparation of the catalysts...17

Figure S6. Experimental procedure for the APR of BS..18

2.5. Analytical methods...19

References...23

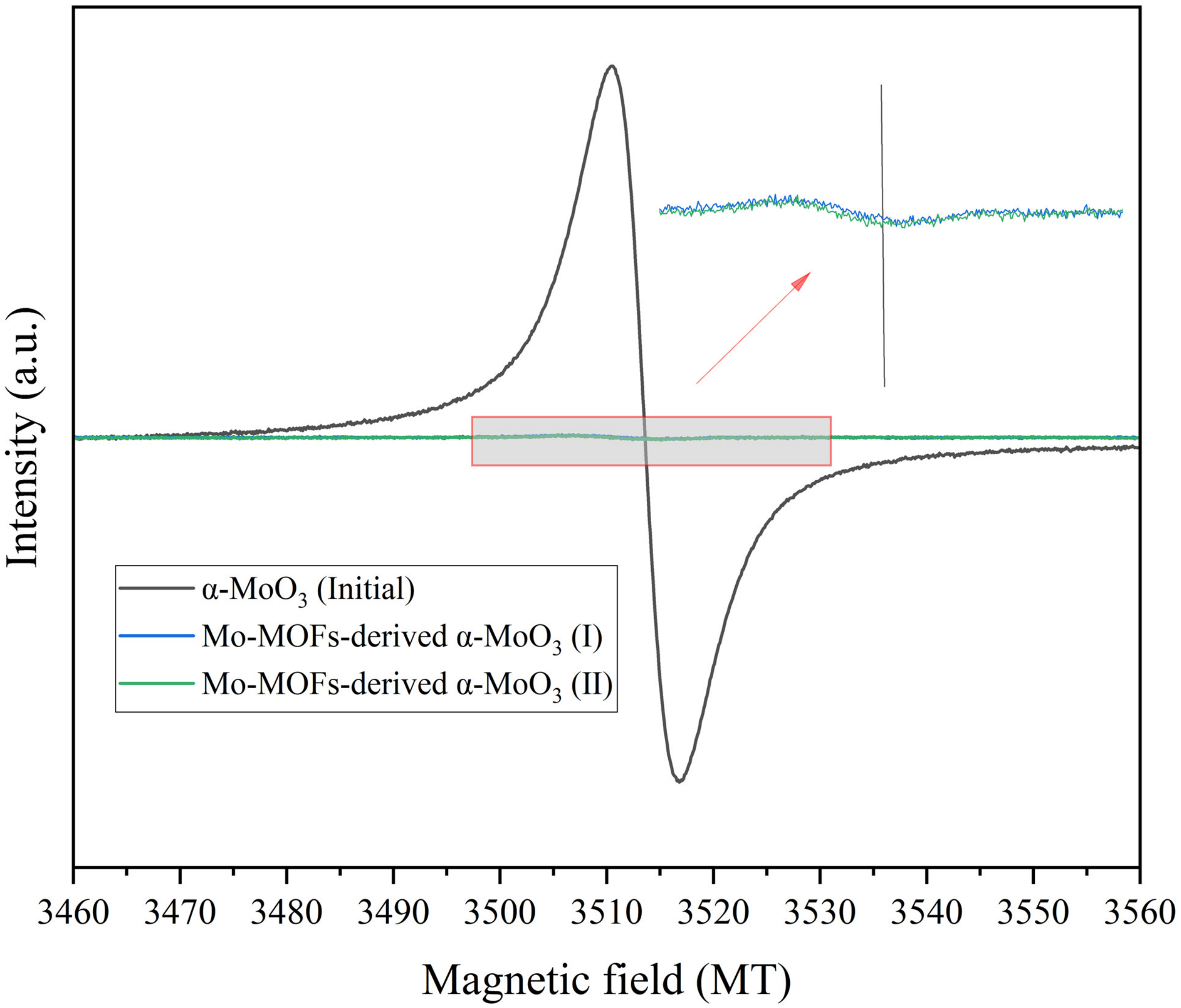

Figure S1. EPR patterns of various catalysts

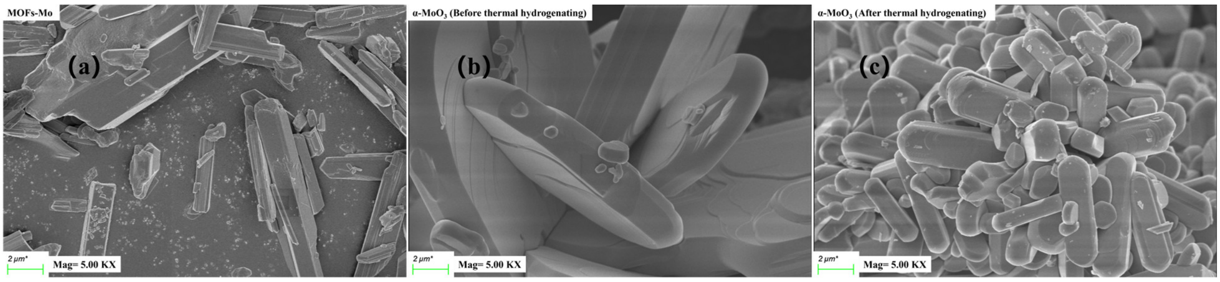

Figure S2. SEM of various catalysts

Table. S1. Distribution of calculated acidic-basic sites and B/L ratio based on Py-IR and CO_2-TPD of catalysts (μmol/g)

Catalyst	B^a	L^a	B/L^a	Total acidity^a	Total basicity^b
α-MoO_3 (Initial)	6.44	47.78	0.13	54.23	69.84
Mo-MOFs-derived α-MoO_3 (I)	4.82	43.03	0.11	47.86	7.05
Mo-MOFs-derived α-MoO_3 (II)	2.89	27.47	0.11	30.37	3.65

*[a] Determined by Py-IR, [b] Determined by CO_2-TPD.

Table. S2. Gases composition, volume and pressure obtained from APR of BS at different fermentation days (Reaction conditions: 225 °C, 30 min, 1 g catalyst, and 50 mL BS)

Fermentation days	Gas composition (vol%)						Gas volume (mL, STP)	Gas pressure (MPa)
	H_2	CO_2	C_2H_4	N_2	CH_4	CO		
0	11.9916	21.8239	0.5990	65.4718	0.0404	0.0733	64.8432	0.1380
2	11.9780	21.4199	0.1932	66.4016	0.0000	0.0073	69.7770	0.1430
4	11.7937	20.8795	0.9302	66.3967	0.0000	0.0000	78.0270	0.1570
6	13.7775	21.0299	0.0000	65.1926	0.0000	0.0000	78.8041	0.1530
8	9.1880	21.4601	0.6767	68.6723	0.0000	0.0029	77.5638	0.1635
10	7.5617	23.7529	2.1500	66.5081	0.0000	0.0273	83.9275	0.1720
12	7.4676	23.1231	0.7336	68.6757	0.0000	0.0000	90.7589	0.1860

Note: The place of decimal point keeps four to better display the composition of gases.

Table. S3. Performance comparison of main characterization and results of APR of biomass-based organic wastewater

No.	Wastewater type	Water quality (Initial)	APR conditions	Reactor type	Catalyst	Water treatment efficiency	H_2 yield	Note	Ref.
1	Fruit juice wastewater without the salinity	COD: 5925 mg/L; pH: 10	219.85 ℃ and 4 h	Batch autoclave	Pt/C	TOC removal: 63%	7.8±0.1 mmol H_2/g COD	-	[2]
	Fruit juice wastewater with the low salinity	COD: 5925 mg/L; pH: 10; Inorganics: 483 mg/L				TOC removal: 63%	6.5±0.1 mmol H_2/g COD	-	
	Fruit juice wastewater with the high salinity	COD: 5925 mg/L; pH: 10; Inorganics: 967 mg/L				TOC removal: 65%	4.2±0.7 mmol H_2/g COD	-	

2	Brewery wastewater with the salinity	TOC: 1646±230 mg/L; COD: 4764±586 mg/L; pH: 11; Phosphate: 15±1 mg/L; Sulfate: 41±4 mg/L; Maleate: 4±1 mg/L	219.85 °C and 4 h	Batch autoclave	Pt/KJB (Carbon blacks, S_{BET}:1350<0.001 m^2/g; Mesopore Volume: 1.59 cm3/g; pH: 10.2)	TOC removal: 75%; COD removal: 78%; pH: 8	12.9±2.3 mmol H_2/g COD	-	[3]
3	Cheese whey wastewater	-	200–240 °C	Fixed bed	Ni-La/Al_2O_3	-	H_2 (8–58 vol.%)	H_2 production is favored at high pressure, and elevated temperature, employing a high amount of catalyst and a concentrated lactose solution.	[4]

								Liquid production is preferential using diluted lactose solutions	
4	Tuna-cooking wastewater with the salinity	TOC: 1895±258 mg/L; COD: 4996±501 mg/L; Chloride: 3815±219 mg/L; Acetate: 1895±321 mg/L; Formate: 10±9 mg/L; Phosphate: 523±22 mg/L; Sulphate: 16±14 mg/L;	199.85 ℃, 4 h	Batch autoclave	Pt/C	TOC and COD removal ranged within 45–60%	Total detected gas: 158±18–268±35 μmol; Total detected gas: 0.5±0.2–8.7±1.6 mol%	-	[5]

		Initial pH: 6.1±0.2							
5	Synthetical brewery wastewater	TOC values around 500 and more than 4000 mg/L; Corresponding COD from about 1500 to more than 11,200 mg/L	199.85 °C, 4 h; 224.85 °C, 4 h	Batch autoclave	Pt/C (different carbon materials)	At the lowest organic load tested ($COD_{initial}$=1531 mg/L), removal values in the range of 80–99% were obtained. Nearly identical removal rates were achieved when the $COD_{initial}$ was increased to 3046 mg/L, while at11,204 mg/L of $COD_{initial}$, the TOC and COD removal decreased to 51–77%	2.8±0.1–15.4±0.2 mmol H_2/g COD	The highest removal of TOC and COD was observed for catalysts supported on highly mesoporous carbon blacks with virtually no microporosity and high pH slurry	[6]
6	Real biogas slurry (RBS)	NPOC 19815±77.78 mg/L; TN 9000±848.53 mg/L;	225 °C, 30 min	Batch autoclave	α-MoO_3 nanosheets	NPOC, COD, and TN reached 79.45%, 87.82% and 44.78%, respectively	4.91 $mL_{hydrogen}/mL_{RBW}$	-	[7]

COD	63600±2262.74	mg/L

*Where TOC, COD, NPOC, and TN represented the total organic carbon, chemical oxygen demand, non-purgeable organic carbon, and total nitrogen, respective

Table. S4. The volume of BS after APR with different fermentation days (Reaction conditions: 225 °C, 30 min, 1 g catalyst, and 50 mL BS)

Fermentation days	Volume (mL)
0	48.00
2	46.00
4	45.00
6	43.00
8	47.50
10	46.00
12	46.00

*Initial volume was 50 mL

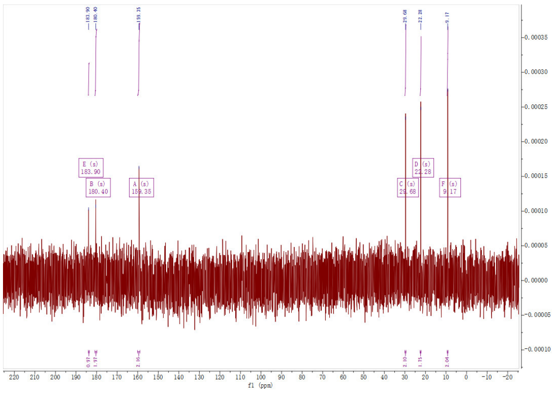

0 (Initial)

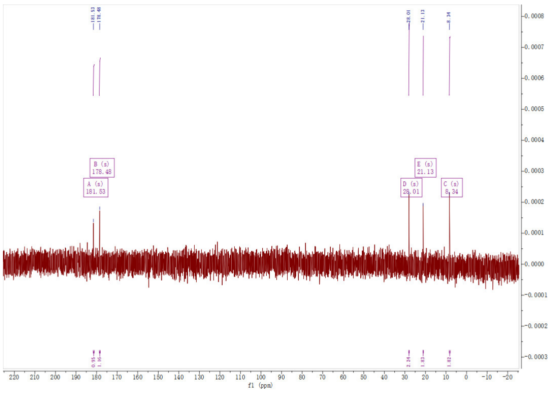

0 (After APR)

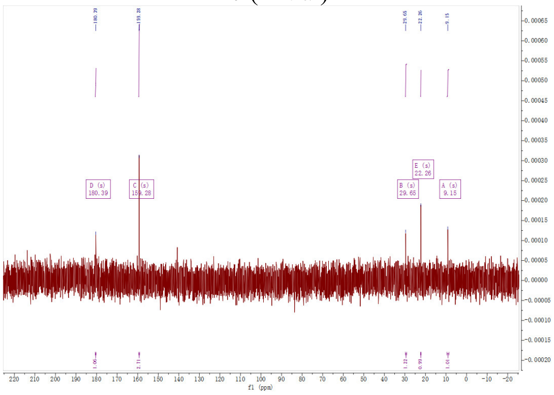

2 (Initial)

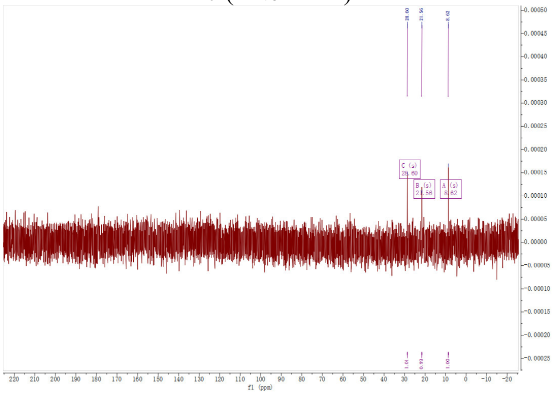

2 (After APR)

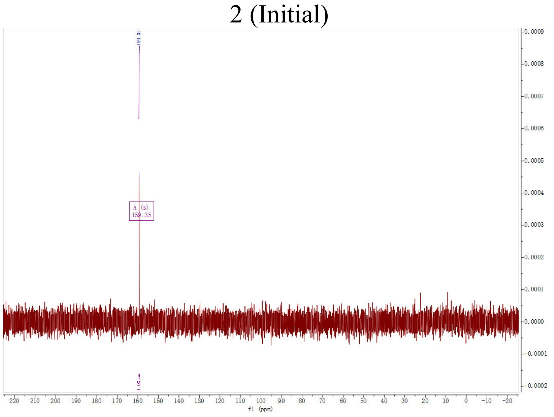

4 (Initial)

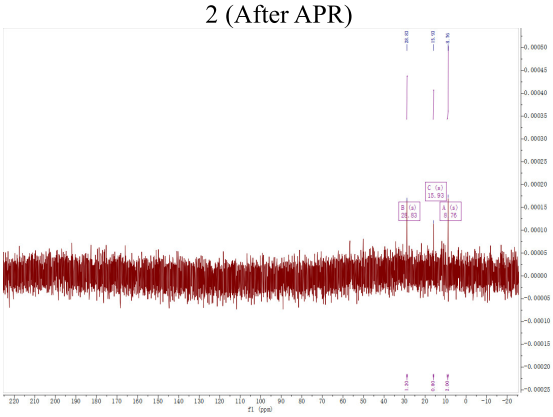

4 (After APR)

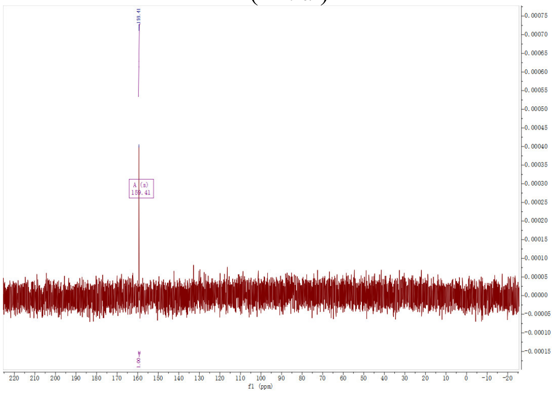

6 (Initial)

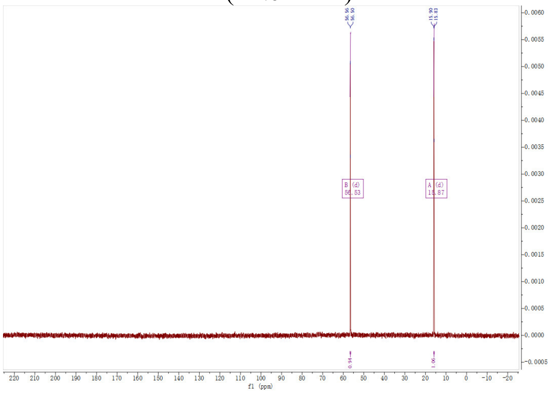

6 (After APR)

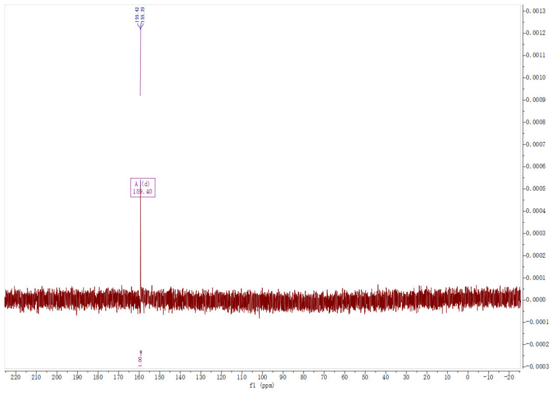

8 (Initial)

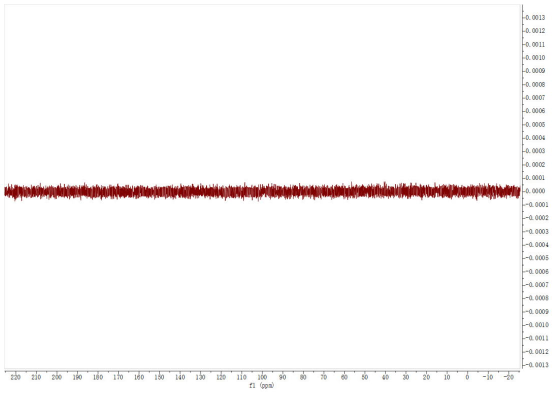

8 (After APR)

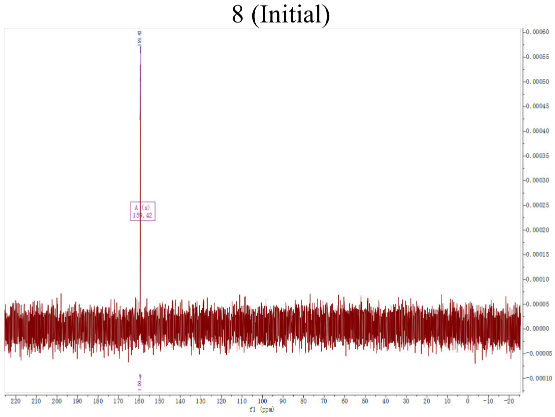

10 (Initial)

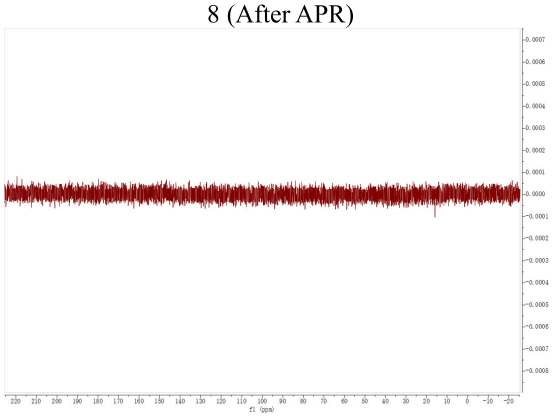

10 (After APR)

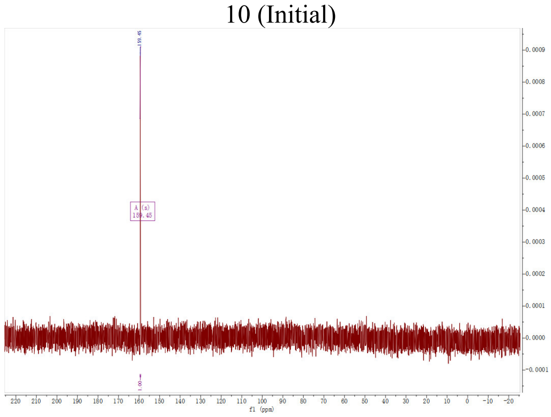

12 (Initial)

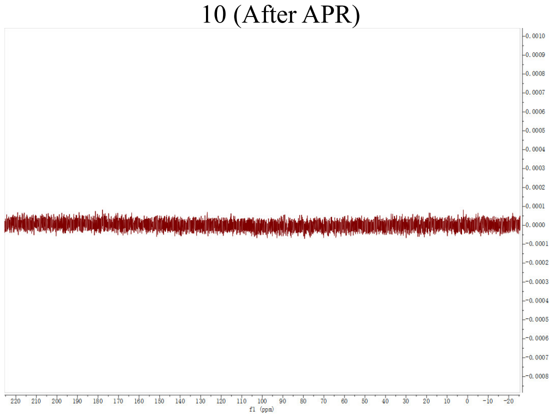

12 (After APR)

Figure S3. ^{13}C-NMR chemical shift assignment ranges and carbon contents of the liquid obtained from gradient BS before and after treatment with Mo-MOFs-derived α-MoO_3 (II) catalysts (Reaction conditions: 225 °C, 30 min, 1 g catalyst, and 50 mL BS)

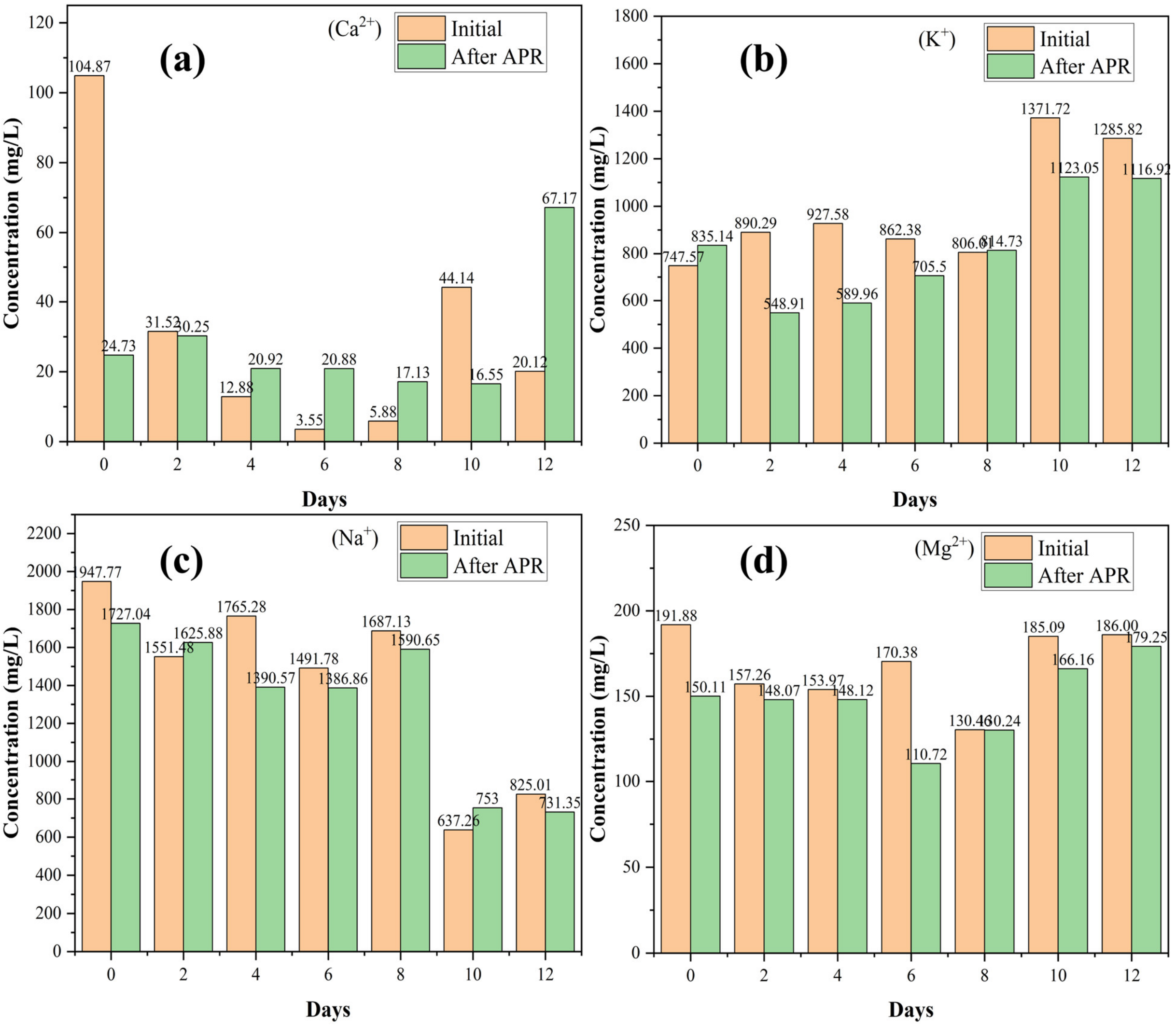

Figure S4. Effect of catalysts on the ion concentration (mg/L) (Reaction conditions: 225 °C, 30 min, 1 g catalyst, and 50 mL BS).

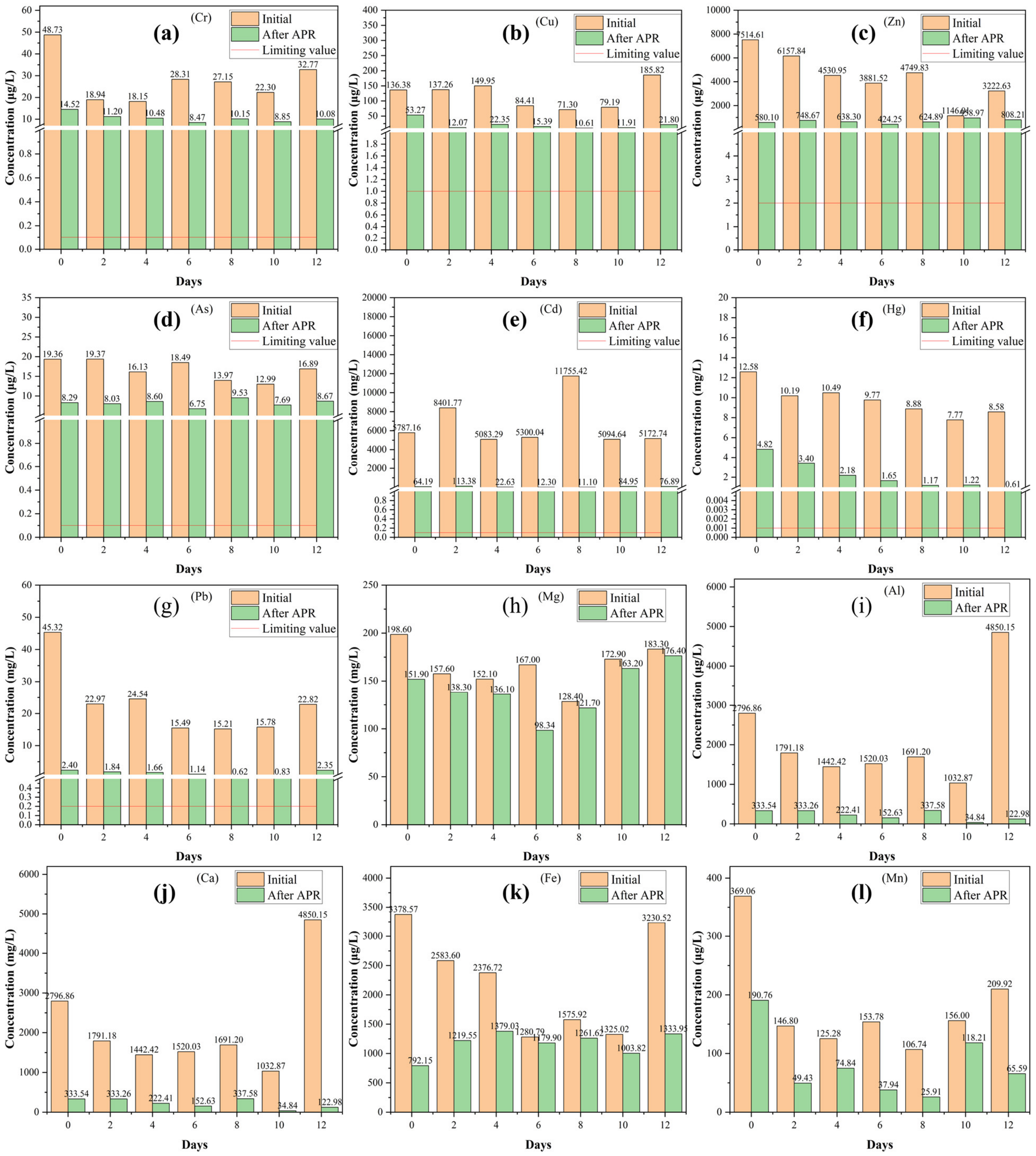

Figure S5. Influence of the treatment without/with catalysts on the heavy metals (μg/L): (a)~(g) the hazardous heavy metals and (h)~(m) the non-hazardous heavy metals (Reaction conditions: 225 °C, 30 min, 1 g catalyst, and 50 mL BS)

2.2. Preparation of the catalysts

Mo-MOFs-derived α-MoO_3 were prepared by the two-step procedure. (1) synthesis of Mo-MOFs-derived α-MoO_3 (I). A typical preparation procedure was the following: 9.49 g imidazole and 20 g α-MoO_3 were dissolved in 100 mL of deionized water. The obtained slurry was transferred to a sealed Teflon-lined autoclave (150 mL) and heated at 150 °C and kept for 24 h to ensure full crystallization. The product was washed 4 times with deionized water and followed by drying at 50 °C to obtain Mo-based MOFs. Then, the as-synthesized product was calcined at 600 °C for 1.5 h under 500 N mL/min air flow to obtain Mo-MOFs-derived α-MoO_3 (I).

The above sample was mixed with 100 mL H_2O_2 (30 wt%) aqueous solutions in a beaker at room temperature and held for 48 h. Then, 100 mL distilled water and 50 mL saccharose aqueous solutions (317.08 g/L) were added to the solution. The mole ratio of C to Mo was 4, and the role of saccharose was to consume excess H_2O_2. This suspension was dried at 80 °C together with stirring until dry. Subsequently, all the samples were dried at 105 °C for 24 h. Then, these samples were calcined at 600 °C at a heating rate of 20 °C/min and held at 600 °C for 1.5 h under 500 N mL/min air flow to obtain Mo-MOFs-derived α-MoO_3 (II).

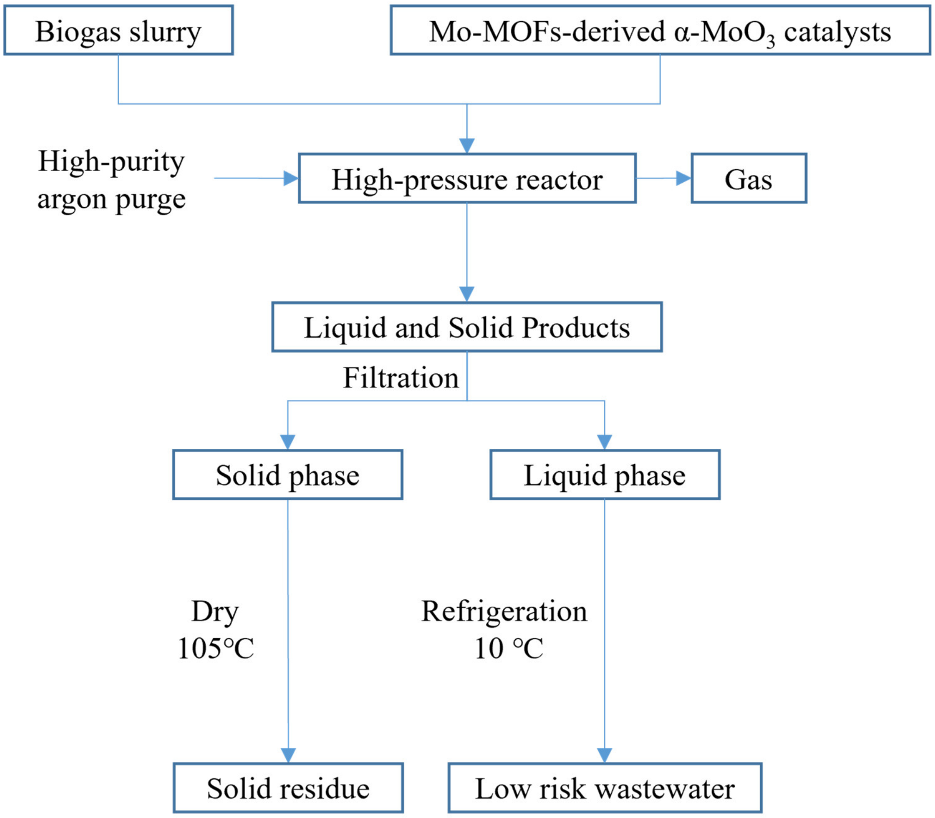

Figure S6. Experimental procedure for the APR of BS

2.5. Analytical methods

The X-ray powder diffraction spectra (XRD) were investigated by PANalytical X'Pert PRO X-ray Diffractometer (PANalytical, Netherlands) with Cu target Kα-ray (operated at 40 kV and 40 mA, with k=0.1541 nm). The scanning was performed with a 2θ angle ranging from 10° to 95° at the rate of 5°/min.

X-ray photoelectron spectroscopy (XPS) analysis was carried out using a Thermo Fisher Scientific ESCALAB 250XiXPS System (America). The binding energy of samples was calibrated using the binding energy of the C 1s peak (284.8 eV) as a reference.

The electron paramagnetic resonance (EPR) tests were carried out in the X-band (9.84 GHz) with 4.00 G modulation amplitude and a magnetic field modulation of 100 kHz using an EPR spectrometer (EMXPlus-10/12, Bruker) at 77 K.

The microstructures of catalysts before and after treatment of BS were examined at 200 kV by scanning electron microscope (SEM) (JEOL, Japan).

Transmission electron microscopy (TEM) with elemental mapping of catalysts before and after use was recorded by a JEM-2100Plus (Japan) at 200 kV accelerating voltage to investigate microstructures. Samples of TEM tests were prepared by dispersing the powder in ethanol, followed by ultrasonication for 30 min.

The surface area and pore size distribution of the catalysts were characterized by a Micrometric Acusorb 2100E apparatus.

The acidic and basic properties of catalysts were characterized by using the temperature-programmed desorption of ammonia and carbon dioxide (NH_3-TPD and CO_2-TPD, CHEMBET-3000 chemisorption instrument). Before each NH_3-TPD run, the catalyst was pretreated under high-purity N_2 at 90 °C for 1 h, then the atmosphere was turned into 10 vol.% NH_3/N_2 with the

flow rate of 30 mL/min for 1 h. Next, the catalyst was flushed with high-purity N_2 to remove physically adsorbed NH_3 on the catalyst surface. Finally, the system of NH_3-TPD was heated from 50 °C to 500 °C at a heating rate of 10 °C/min, and the NH_3 desorption profiles were recorded under N_2 flow by TCD detector. Additionally, CO_2-TPD repeated the above steps, just NH_3 was replaced with CO_2.

Py-IR analysis was conducted on a Thermo Nicolet 6700 FT-IR spectroscopy to characterize the acid properties of catalysts. Before analysis, the catalysts were pretreated at 300 °C for 2 h under vacuum and then cooled down to room temperature to collect a background spectrum. Following the injection of pyridine, the sample cell was saturated for 30 min and then evacuated for 30 min at 150 °C under a vacuum. Finally, the spectrum was collected at room temperature. From the results of Py-IR, quantitative analysis of Lewis and Brønsted acid densities were calculated by the Eqs. (5–6)[1]. The quantitative analysis of Lewis and Brønsted acid densities of catalysts are calculated using the following equations:

C (pyridine on Lewis acid sites) = $1.42 \cdot IA(L) \cdot R^2 / W$ (5)

C (pyridine on Brønsted acid sites) = $1.88 \cdot IA(B) \cdot R^2 / W$ (6)

where C is acid density (mmol/$g_{catalyst}$), and IA (L, B) is integrated absorbance of Lewis and Brønsted bands at 1448 and 1547 cm^{-1} respectively. R and W are the radius of the wafer disk (cm) and the weight of the disk (mg), respectively. These two equations are assumed that all Brønsted acid sites processed the same integrated molar extinction coefficient (integrated molar extinction coefficient: 2.22 and 1.67 cm/μmol for Lewis and Brønsted acid sites respectively).

The hydrogen reduction of catalysts was conducted on temperature-programmed oxidation of oxygen (H_2-TPO, CHEMBET-3000 chemisorption instrument) to characterize the reducibility of catalysts. Before each test for H_2-TPR, the catalyst was pretreated under high-purity N_2 at 30 °C

for 1 h, then heated from 50 °C to 500 °C at a heating rate of 10 °C/ min under 12 vol.% H_2/N_2 atmosphere with a flow rate of 30 mL/min in the dark or under simulated solar irradiation. The illumination condition was achieved by equipping the furnace with a small window on one side and 300 W Xe lamps on the outside. The consumption of H_2 was detected using a thermal conductivity detector (TCD).

The carbon deposition of catalysts was characterized by using the temperature-programmed oxidation of oxygen (O_2-TPO, CHEMBET-3000 chemisorption instrument). For the O_2-TPO experiment, 20 mg of the samples were pretreated at 30 °C for 1 h under high-purity Ar flow, then exposed to 40 vol.% O_2/Ar at a flow rate of 30 mL/min. O_2-TPO profile was recorded from 50 °C to 900 °C at a heating rate of 10 °C/min. Gas compositions were analyzed by a HIDEN QIC-20 mass spectrometer.

The COD, AN, and TN of BS before and after treatment were measured by apparatus (DRB200/DR900, HACH, American). The NPOC of BS before and after treatment was measured by TOC-L CPH/CPN (SHIMADZU, Japan).

The volume of BS before and after treatment was measured by a 50 mL volumetric cylinder.

The pH of BS before and after treatment was examined using a pH meter (PHS-3C, INESA Scientific Instrument Co., Ltd, Shanghai, China).

The salinity of BS before and after treatment was examined using a salinity meter (METTLER TOLEDO, FiveEasy Plus, China).

The gases products of H_2, CO, CO_2, N_2, and other hydrocarbons were analyzed by a GC/FID/TCD (Agilent, 7890A) using a 5A molecular sieve column (2 m) and Porapak Q columns (3 m). The temperature was increased from 50 °C to 280 °C at a rate of 6 °C/min and then held at 280 °C for 5 min. The carrier gas was helium, and its flow rate was 2 mL/min.

The 400 MHz liquid nuclear magnetic resonance (NMR) measurements were performed on ^{13}C-NMR spectra (AV 400, Bruker Inc., Switzerland) to detect the functional groups of BS before and after treatment. The samples of NMR were prepared by dissolving weighed -50 mg of BS before and after treatment. Chemical shifts were referenced externally to the DIMETHYL SULFOXIDE-D6 (Cambridge Isotopc laboratories, Inc., Beijing, China) aqueous solution at 0 ppm. In spectral analysis, the integration values of given peaks were compared to the total area of all peaks, resulting in mol% units.

Anions and NH_4^+ in BS before and after treatment were carried out using the ion chromatography instrument (ICS-1100, Thermo Dionex, USA). K^+, Ca^{2+}, Na^+, $Mg^{2+,}$ and heavy metals in BS and catalysts before and after treatment were analyzed by ICP-MS (iCAP Q, Thermo, Waltham, USA).

References

[1] H.S. Kim, J.B. Cook, H. Lin, J.S. Ko, S.H. Tolbert, V. Ozolins, B. Dunn, Oxygen vacancies enhance pseudocapacitive charge storage properties of MoO_{3-x}. Nature Mater. 16 (2017) 454–460. https://doi.org/10.1038/nmat4810.

[2] B. Saenz de Miera, A.S. Oliveira, J.A. Baeza, L. Calvo, J.J. Rodriguez, M.A. Gilarranz, Treatment and valorisation of fruit juice wastewater by aqueous phase reforming: Effect of pH, organic load and salinity. J. Clean. Prod. 252 (2020) 119849. https://doi.org/10.1016/j.jclepro.2019.119849.

[3] A.S. Oliveira, J.A. Baeza, D. Garcia, B. Saenz de Miera, L. Calvo, J.J. Rodriguez, M.A. Gilarranz, Effect of basicity in the aqueous phase reforming of brewery wastewater for H_2 production. Renew. Energ. 148 (2020) 889–896. https://doi.org/10.1016/j.renene.2019.10.173.

[4] J. Remón, J. Ruiz, M. Oliva, L. García, J. Arauzo, Cheese whey valorisation: Production of valuable gaseous and liquid chemicals from lactose by aqueous phase reforming. Energ. Convers. Manage. 124 (2016) 453–469. http://dx.doi.org/10.1016/j.enconman.2016.07.

[5] A.S. Oliveira, J.A. Baeza, L. Calvo, N. Alonso-Morales, F. Heras, J. Lemus, J.J. Rodrigueza, M.A. Gilarranz, Exploration of the treatment of fish-canning industry effluents by aqueous-phase reforming using Pt/C catalysts. Environ. Sci-Wat. Res. 4 (2018) 1979-1987. https://doi.org/10.1039/C8EW00414E.

[6] A.S. Oliveira, J.A. Baeza, L. Calvo, N. Alonso-Morales, F. Heras, J.J. Rodriguez, M.A. Gilarranz, Production of hydrogen from brewery wastewater by aqueous phase reforming with Pt/C catalysts. Appl. Catal. B-Environ. 245 (2019) 367–375. https://doi.org/10.1016/j.apcatb.2018.12.061.

[7] J. Wang, Z.B. Liu, R. Liang, B.B. Yan, J.Y. Tao, H. Su, G.Y. Chen, Aqueous phase reforming of distiller's grain derived biogas Plant wastewater over α-MoO_3 nanosheets.

Chem. Eng. J. 430 (2022) 132735. https://doi.org/10.1016/j.cej.2021.132735.
